# Making the Public Understand

**Published:** 1920-09-11

**Authors:** 


*To the Editor of* THE HOSPITAL.

### Making the Public Understand.

*To the Editor of* THE HOSPITAL.

SIR,—I am grateful to the writer of "The Way of Attainment" for the courtesy of a reply. I should think it is now generally agreed that the Council of the College of Nursing and the General Nursing Council are "our leaders." Would it not seem desirable that publicity should be given to the proceedings of the former, inasmuch as it appears to be given to the latter? We have been crusading with propaganda work for the past fifty years or thereabouts, and the general public are not much the wiser of the combined effort. At the present moment with the public a nurse is a nurse if she wears uniform, whether she be cottage, village, or hospital-trained, matron, or matron-in-chief—not that the latter would mind passing for the former, and it would be a great honour for the former to pass for the latter; but apart from this, and also from what I have already stated in my former note, the chief point is, it would be a tremendous help to those struggling with the organisation of public work and in trying to get cosmos out of chaos. To my thinking we have arrived at the time that it is both needful and desirable to get as much publicity as possible to back up and support those in the struggle. It is only those who have laid or are now laying the foundation of new work who can appreciate this point (the importance of educating the public).

I do not think it is at all a matter for financial discussion. Editors are only too glad to get valuable information, especially on new subjects, and with the new work this would appear to be the time to seize the opportunity; also I feel sure the Council of the College will be alive to its importance. If we could get the account of the proceedings of the two Councils in our London press we would not trouble with the local centres just yet. What is needed is information from the main bodies through the main channels to the public.

I do not see why we should be gloomy about this matter. A keen sense of fun and humour above all things is uplifting in these troublous times, but I should not put down their precious qualities to frivolity—there is a line of demarcation—and I should certainly let them flourish as a true antidote to the poison of pessimism: it comes as a breath of the high hills.—Yours faithfully,

A COLLEGE MEMBER.

